# TAC3/TACR3 System Function in the Catadromous Migration Teleost, *Anguilla japonica*

**DOI:** 10.3389/fendo.2022.848808

**Published:** 2022-07-22

**Authors:** Chenpeng Zuo, Likang Lyu, Wenhui Zou, Haishen Wen, Yun Li, Xin Qi

**Affiliations:** ^1^ Key Laboratory of Mariculture, Ministry of Education, Ocean University of China, Qingdao, China; ^2^ College of Ocean, Key Laboratory of Tropical Biological Resources of Ministry of Education, Hainan University, Haikou, China

**Keywords:** tachykinins, neurokinin B, reproductive regulation, ovary development, Japanese eel

## Abstract

Neurokinin B (NKB), a member of the tachykinin (TAC) family, plays important roles in mammalian neuropeptide secretion in related to reproduction. However, its potential role in spawning migration teleost is less clear. In the present study, Japanese eel (*Anguilla japonica*) was employed to study the performance of NKB in regulating reproduction. Results showed that two *tac3* and one *tacr3* genes were identified in Japanese eel. Sequence analysis showed that two *tac3* transcripts, *tac3a* and *tac3b*, encode four NKBs: NKBa-13, NKBa-10, NKBb-13, and NKBb-10. However, compared with other species, a mutation caused early termination of TACR3 protein was confirmed, leading to the loss of the 35 amino acid (aa) C-terminal of the receptor. Expression analysis in different tissues showed that both *tac3a* and *tac3b* mRNAs were highly expressed in the brain. *In situ* hybridization localized both *tac3a* and *tac3b* mRNAs to several brain regions, mainly in the telencephalon and hypothalamus. Because of the mutation in TACR3 of Japanese eel, we further analyzed whether it could activate the downstream signaling pathway. Luciferase assay results showed the negative regulation of cAMP Response Element (CRE) and Sterol Response Element (SRE) signal pathways by Japanese eel NKBs. Intraperitoneal injection of four different NKB mature peptides at 100 ng/g had negative effect on either *gnrh* or *gth* gene expression. However, the high concentration of NKBa-10 and NKBb-13 (1,000 ng/g) upregulated *mgnrh* and *fshb* or *lhb* expression level significantly, which may be mediated by other receptors. In general, the NKBs/NK3Rs system has important functions in regulating eel puberty onset.

## Introduction

Puberty refers to the developmental transition to a mature, reproductive state in mammals (1). In rodents, the first external sign of pubertal development is vaginal opening, although hormonal changes precede this (2). In fish, puberty is the developmental period during which an individual becomes capable of reproducing sexually for the first time and implies a functional competence of the brain–pituitary–gonad axis (3–5). It starts sometimes after sex differentiation and is associated with the initiation of germ cell maturation and full functional differentiation of the germ cell–supporting somatic cells of the gonads and culminates in the first spermiation and sperm hydration or ovulation (6).

Japanese eel, *Anguilla japonica*, is one of the most important species of the aquaculture industry of East Asia. However, because of the catadromous migration reproductive character, the aquaculture industry is limited by the natural resources of glass eels from the ocean. Artificial reproduction studies of elopomorphes started in early 1930s, while until now, although great process has been made (7), the maturation rate of parental eels, survival rate of fertilized eggs, growth speed of hatched larva are still far beyond industrial application, especially the extremely high cost in raring hatched larva, limited this industry to the new horizon. The elopomorphes have a complex migratory life cycle with the occurrence of two metamorphoses (8). The first one is when leptocephali larvae metamorphose into glass eels at the time of their arrival over the continental shelf, at the end of a long oceanic journey, after which, glass eels become “yellow” eels; the second metamorphosis is termed “silvering (transition from yellow to silver eel)”, after years of growing in freshwater, the yellow eels stop growth and start the downstream migration toward the area for breeding in the ocean (9). The silvering of eels was suggested to be considered as a pubertal development step, according to hormonal profiles and experimental results on the gonadotropic axis (10). It is believed that, at the silver stage, eels are blocked at a prepubertal stage (11), but chronic administration of carp or salmon pituitary extract (gonadotropic treatment) is able to induce sexual maturation (12, 13).

The neurokinin B (NKB), coded by the *tac3* gene (*tac2* gene in rodents), is an important member of tachykinin family. NKB, kisspeptin, and dynorphin are co-expressed in the arcuate nucleus and play an important role in the regulation of GnRH secretion (14–17). The NKB signal was important in reproduction especially puberty of human. Mutations in the genes encoding NKB (*tac3*) or its receptor NK3R (*tacr3*) lead to hypogonadotropic hypogonadism—a disease characterized by the failure of sexual maturation, impaired gametogenesis, and infertility, indicating that the NKB/NK3R system is indispensable for human reproduction (18, 19). In monkeys (*Macaca mulatta*), transient activation of NK3R stimulates GnRH release, but repeated stimulation of this pathway does not induce GnRH pulsatile release (20). In addition, injecting antagonists of NK3R could inhibit LH secretion levels in ovariectomized rats (*Rattus norvegicus*) (21). Taken together, in addition to its role in the downstream part of the pubertal mechanism (GnRH pulse generator), NKB signal may also be involved in the upstream part of the central pubertal system.

In teleost, NKB/NK3R signaling pathway has been characterized in zebrafish (*Danio rerio*) (22), goldfish (*Carassius auratus*) (23), tilapia (*Oreochromis mossambicus*) (24), spotted sea bass (*Lateolabrax maculatus*) (25) and carp (*Ctenopharyngodon idella*) (26). Previously, using goldfish as a modal, we have confirmed the NKB function in regulating GnRH and GtH expression both *in vivo* and *in vitro* (23). Furthermore, we have also demonstrated that NKB could act directly on the ovary to enhance the 17β-estradiol (E_2_) synthesis and release and, therefore, the growth of oocytes (27). In the present study, we used Japanese eel, whose ovary development is blocked at the prepubertal stage, as a model to further investigate the role of NKBs in fish puberty onset. cDNA for *tac3a*, *tac3b*, and *tacr3s* were characterized and cloned, and the distribution of *tac3s* in different tissues was determined. Because *tac3s* mRNA was highly expressed in the brain, we further detected the location of both genes in brain regions of the eel. Interestingly, mutation caused early termination of NK3R protein translation was found in all tested samples. To confirm whether the mutated NK3R could active the downstream signaling, using luciferase assay, we confirmed that NKB of Japanese eel could not activate neither CRE nor SRE signaling pathway. Finally, to evaluate whether NKB can still regulate the reproductive functions by receptors of other genes, we measured two Japanese eel GnRHs that were homologous to mammalian or chicken GnRH (namely, *mgnrh* and *cgnrh*, respectively), *fshb* and *lhb* gene expression by real-time PCR in eels injected with NKBs. These novel findings demonstrated that the NKBs/NK3Rs system has important functions in regulating eel puberty onset.

## Materials and methods

### Animals

Female Japanese eels with body weight (BW) of 1.0 ± 0.2kg were obtained from Guangzhou, Zhuhai (Guangdong), and Qingdao (Shandong), China, and allowed to acclimatize for 7 days in tanks with a photoperiod of 14L:10D and temperature of 25°C. Zebrafish were obtained from an ornamental fish market in Qingdao, China. The animals were fed with commercial diet without any supplemental hormones. All animal experiments were conducted in accordance with the guidelines and approval of the Animal Research and Ethics Committees of Yat-Sen University and the Ocean University of China.

### Cloning and Sequence Analysis of *tac3s/tacr3s* Genes

Total RNA from more than 80 Japanese eel brains and ovaries and three zebrafish brains were prepared by using TRIzol (Invitrogen, USA). One microgram of isolated RNA was used to synthesize cDNA using the ReverTra Ace-a First-Strand cDNA Synthesis Kit (TOYOBO, Japan). To amplify the cDNA fragments of the eel *tac3a*, *tac3b*, and *tacr3*, PCR primers were designed on the basis of the nucleotide sequences of searched from the genome data. Full-length cDNA sequences encoding these genes were obtained by the 5′ and 3′ rapid amplification of cDNA ends (RACE) with several gene-specific primers (**Table 1**). The *tacr3a1* sequence of zebrafish was obtained from National Center for Biotechnology Information (NCBI) and cloned. The primers used were listed in **Table 1**. For all PCR reactions, amplifications were performed as follows: denaturation at 94°C for 5 min, followed by 40 cycles at 94°C for 20 s, 55°C–60°C for 20 s, and 72°C for 2 min. The reaction ended with a final extension at 72°C for 5 min. The amplification products were purified using the E.Z.N.A. Gel Extraction Kit (Omega BioTek, USA) and subcloned into the pGEM-T vector (Promega, USA). Three different positive clones were sequenced on an ABI 3700 sequencer (Applied Biosystems, USA). The putative amino acid (aa) sequences were predicted using the BioEdit software, and the putative seven-transmembrane domains were predicted using the TMHMM Server v. 2.0 (http://www.cbs.dtu.dk/services/TMHMM-2.0/). The NKBs aa sequences were aligned with Clustal W 1.81 (28). The protein phylogenetic analysis was conducted with MEGA 6.0 using the neighbor-joining method.

**Table 1:** Primers for RACE, gene clone, qPCR, and ISH.

Primers	Sequence (5′-3′)
TAC3a-race-F1	AACGGTATAGATTATGACAGC
TAC3a-race-F2	GCCTTATGGGTAGAAGAAGCA
TAC3a-race-R1	GAATAGGTGGACCTGCCTTG
TAC3a-race-R2	ACTCTGAACTCCTCCGACCC
TAC3b-race-F1	ATGACACCTTTGTAGGGCTGAT
TAC3b-race-F2	CTGATGGGCAGAAGAAGT
TAC3b-race-R1	TGGATTCTGAACTCCTCC
TAC3b-race-R2	CCATTAGTCCCACGAAGAT
TAC3R-race-F1	GAAGCCCAGACTGTCAGCCACG
TAC3R-race-F2	CGAAGGACCCTCTGCTATGTG
TAC3R-race-R1	GTCCATGGTAATTGTCTGAC
TAC3R-race-R2	ACACCAGCACAGTCACAA
NUP	AAGCAGTGGTATCAACGCAGAGT
UPM (long)	CTAATACGACTCACTATAGGGCAAGCAGTGGTATCAACGCAGAGT
UPM (short)	CTAATACGACTCACTATAGGGC
TAC3a-ORF-F	CAGGAAAACAAAAGAGACGATG
TAC3a-ORF-R	GCTGCTGGGACTGGCCTA
TAC3b-ORF-F	CGGCCAGAAAGAGACGATG
TAC3b-ORF-R	AGGGCGAAAAGCTGGTCTTA
TAC3R-ORF-F	ATGGAAGCATCAAACAGCACAT
TAC3R-ORF-R	CTACCTGCTATTGAGGCAGCA
TAC3a-real-F	AAGAGCAGGAACCGCACAAGC
TAC3a-real-R	CCCATCAGGCCAACAAAGAAA
TAC3b-real-F	TATGACACCTTTGTAGGGCTGAT
TAC3b-real-R	AAATGGCTCCTCTTCCCTGTG
TAC3R-real-F	ATCGTCTGTATCTGGGCACTG
TAC3R-real-R	AATTGTCTGACGAATCTCCTGG
18S-real-F	CATTGGAGGGCAAGTCTGGTG
18S-real-R	GCGGGACACTCAGCTAAGAGC
EF1α-real-F	GGTATGGTGGTGACCTTTGCC
EF1α-real-R	CTACGTTGCCACGACGGATTT
β-actin-real-F	TGCGTGACATCAAGGAGAAGC
β-actin-real-R	ATTCCGCAGGACTCCATACCC
mGnRH-real-F	GCAATCCCTCTTCGTCACTC
mGnRH-real-R	CAGCCAGATTTGCCTGTAAG
cGnRH-real-F	CAGGTATTGGAAGAGATAAAGC
cGnRH-real-R	GCTGTATGCTATCCCCTCCT
LH-real-F	GTCCAAAATGTCTGGTGTTC
LH-real-R	GCACAGGTTACAGTCGCAGC
FSH-real-F	CGTGGAGAATGAAGAATGCG
FSH-real-R	CAGGGTAGGTGAAGTGGAGG
TAC3a-ISH-F	CGCATTTAGGTGACACTATAGAAGCGGTTGGCGATCCTGTCCCTTA
TAC3a-ISH-R	CCGTAATACGACTCACTATAGGGAGACAAAGAAGAATCCGCCTGTCCG
TAC3b-ISH-F	CGCATTTAGGTGACACTATAGAAGCGTACTTGGTGTTCGCGATCCT
TAC3b-ISH-R	CCGTAATACGACTCACTATAGGGAGACAAAATGGCTCCTCTTCCCTGTG
Zebrafish-F	TCCAGTTGTCACACAGAGCG
Zebrafish-R	GTGTTTGTCATGATCGCTTGC

### RNA Extraction, Reverse Transcription, and qPCR

Total RNA was extracted using TRIzol reagent (Life Technologies China, Inc.). The amount and purity of the RNA were determined on a NanoDrop 2000 spectrophotometer (ThermoFisher Scientific). One microgram of isolated RNA was used to synthesize first-strand cDNAs using the ReverTra Ace-a First-strand cDNA Synthesis Kit (TOYOBO, Japan). Real-time PCR was performed on a Roche LightCycler 480 using the SYBR Green I Kit (TOYOBO, Japan) according to the manufacturer’s instructions. Elongation factor-1α, β-actin, and 18s were used as internal controls. Relative mRNA levels were determined using the standard ΔΔcycle threshold method.

### *In Situ* Hybridization

The *in situ* hybridization was performed as we previously reported (23, 29). The brains of five female Japanese eels were removed and fixed in buffered 4% paraformaldehyde for 24 h and then embedded in paraffin. Seven-micron-thick sections were cut for ISH. The sections were pasted onto aminopropylsilane-treated glass slides and dried in an oven at 37°C. Sense and antisense digoxigenin (DIG)–labeled riboprobes about 300–400 bp in length were synthesized from the open reading frames (ORFs) of Japanese eels tac3s genes using a DIG RNA Labeling Kit (Roche Diagnostics, Mannheim, Germany). The sections were briefly rehydrated by a graded series of ethanol solutions (100% to 80%) after being cleared in xylene, permeabilized with 0.8 M HCl for 10 min followed by proteinase K (10 µg/ml) digestion for 2 min, washed in 1× Phosphate Buffered Saline (PBS) for 10 min, then washed in 2×Sodium Citrate Buffer (SSC) for 10 min, prehybridized at 55^◦^C for 1 h, and hybridized with DIG-labeled riboprobes diluted in hybridization buffer at 55°C overnight in a wet box (25). After hybridization, the sections were washed in grade series of Sodium Citrate Buffer (SSC) and Phosphate Buffered Saline (PBS) solution and blocked with blocking reagent (Roche Diagnostics). DIG was detected with an alkaline phosphatase-conjugated anti-DIG antibody (Roche Diagnostics; diluted 1:3,000), and chromogenic development was conducted with an Nitro-Blue-Tetrazolium/5-bromo-4-chloro-3-inodlyl-phosphate (NBT/BCIP) stock solution (Roche Diagnostics).

### Peptide Synthesis and Preparation

On the basis of the result that we got of the eel TAC3A and TAC3B, peptides corresponding to the eel NKB peptides (SGTGLSATLPQRF-NH2) were synthesized by GL Biochem (Shanghai, China). The purity of the synthesized peptides was >95% as determined by analytical HPLC. Eel peptides were dissolved into DMSO and diluted to the desired concentration in saline (0.7% NaCl) for *in vivo* injection experiments.

### *In vivo* Injection of TAC in Japanese Eel

A total of 45 healthy Japanese eels with similar BW were selected and divided into nine groups. Five eels in each group were kept in different aquariums, and the aquariums were numbered. The control group was injected with 0.2 ml of saline with 0.1% Dimethyl sulfoxide (DMSO), whereas the treatment groups were injected intraperitoneally with high concentration (1,000 ng/g BW) and low concentration (100 ng/g BW).

### Cell Culture and Co-transfection

The *tacr3* cDNAs of Japanese eel and zebrafish were subcloned into the pcDNA3.1 expression vector (Invitrogen, USA). The COS-7 cells and 293T cells were maintained at 37°C in Dulbecco's Modified Eagle Medium (DMEM) containing 10% fetal bovine serum (Gibco, USA). Twenty hours before transfection, 1 × 10^5^ cells per well were seeded into 24-well tissue culture plates. A total of 500 ng of SRE-Luc or CRE-Luc reporter plasmid (30), 300 ng of pcDNA3.1-*tacr3* of Japanese eel, and 50 ng of pRL-CMV containing the Renilla luciferase reporter gene (31) were co-transfected into the COS-7 cells in 500 ml of serum-free medium using Lipofectamine reagent (Invitrogen, USA). Six hours after transfection, cells were incubated with vehicle or various (from 10^−10^ to 10^−6^ M) concentrations of NKBa-10, NKBa-13, NKBb-10, and NKBb-13 for a further 24 h (stimulated with 1 × 10^−5^ M FSK (Sigma, USA) for CRE promoter). A total of 250 ng of SRE-Luc or CRE-Luc reporter plasmid, 200 ng of pcDNA3.1-*tacr3a1* of zebrafish, and 50 ng of TK containing the Renilla luciferase reporter gene (31) were co-transfected into the 293T cells in 500 ml of serum-free medium using Lipofectamine 3000 reagent (Invitrogen, USA). Six hours after transfection, cells were incubated with vehicle or various (from 10^−9^ to 10^−7^ M) concentrations of NKBa-10 for a further 36 h. Cells were harvested, and luminescence was measured by the Dual Luciferase Kit (Promega, USA).

### Statistical Analysis

All data were expressed as the mean ± S.E.M, and the number of samples is indicated in the figure legends. Statistical significance was determined using a one-way ANOVA followed by Dunnett’s test for multiple rage comparison. A Student’s t-test was used for comparison between two groups. Statistical significance was defined as *P <* 0.05.

## Results

### Identification and Synteny Analysis of the *tac3* and *tacr3* Genes in Japanese Eel

**Figure 1:**
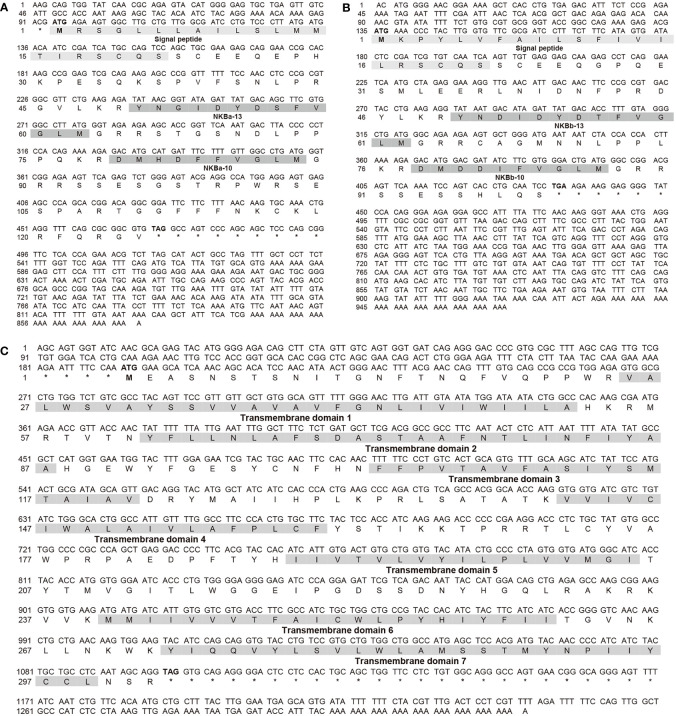
Nucleotide sequences and deduced amino acid sequences of *tac3a* **(A)** and *tac3b* **(B)** in Japanese eel. The signal peptides and NKB peptides were shaded, and their names under the corresponding sequence. Nucleotide sequences and deduced amino acid sequences of *tacr3* **(C)** in Japanese eel. The transmembrane domains were numbered under the corresponding sequence, and their sequence compositions are shaded.

**Figure 2:**
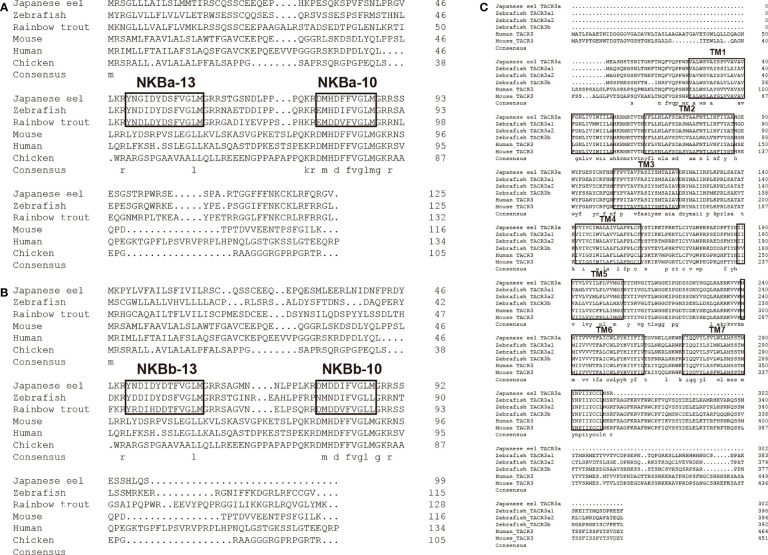
Comparison of the amino acid sequences of Japanese eel *tac3a* **(A)** and *tac3b* **(B)** precursors from different species. The boxed letters indicated the sequence of mature NKB peptides in the detected species. Comparison of the amino acid sequences of Japanese eel *tacr3* **(C)** precursors from different species. The boxed letters indicated the sequence of transmembrane domains in the detected species.

Two *tac3* genes and one *tacr3* gene from the Japanese eel were cloned, named *tac3a* (OL804257), *tac3b* (OL804258), and *tacr3* (ON402757). The ORF of *tac3a* is 375 bp, coding a 125-aa precursor with a predicted signal peptide of 21 aa (**Figure 1A**). The ORF of *tac3b* is 297 bp, coding a 99-aa precursor with a predicted signal peptide of 21 aa (**Figure 1B**). In addition, the ORF of *tac3r* is 909 bp coding a 302-aa G protein–coupled receptor (GPCR) with seven transmembrane domains (**Figure 1C**). Sequence analysis showed that each precursor contains two putative peptides. The four Japanese eel tachykinin peptides were designated NKBa-13, NKBa-10, NKBb-13, and NKBb-10 according to their length. Sequence analysis showed that a common C-terminal sequence (FVGLM) was conversed in all NKB-10 and NKB-13 in teleost. Sequence alignment of TAC3 precursor in teleost showed that NKBa-10 and NKBa-13 were better conserved, whereas NKBb-10 and NKBb-13 had lower similarity in teleost (**Figures 2A, B**). Sequence comparison analysis revealed the TACR3 had a mutation of adenine at the 908th position, resulting an early termination of the GPCR translation (**Figure 2C**).

**Figure 3:**
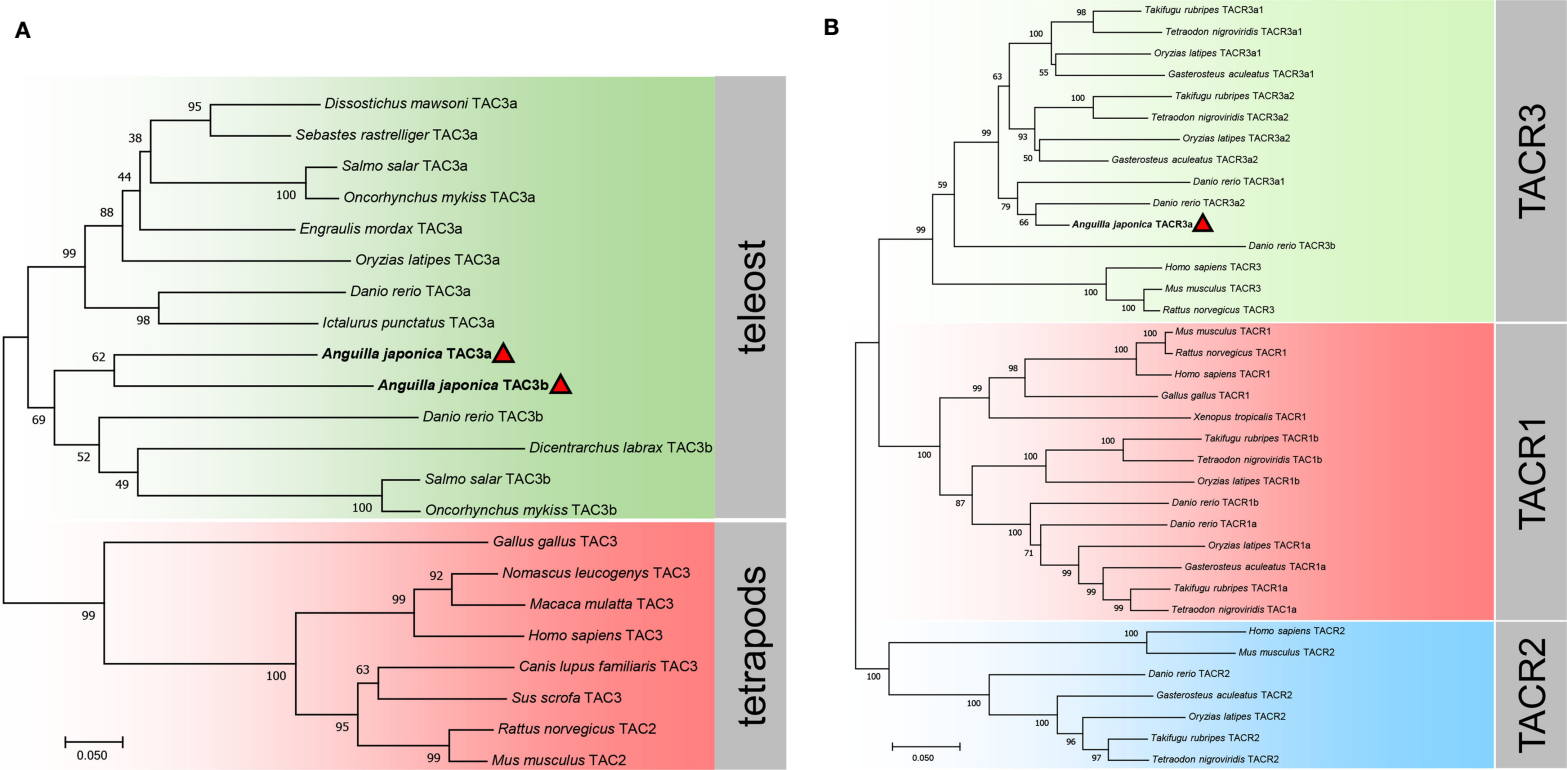
Phylogenetic analysis of the tac3 **(A)** and tacr **(B)** gene family in vertebrates. The phylogenetic trees were constructed by MEGA 7 using the neighbor-joining method. Data were resampled with 1,000 bootstrap replicates. The accession numbers of each sequence are *Dissostichus mawsoni* tac3a (DAA35156.1), *Sebastes rastrelliger* tac3a (XP_037630002.1), *Salmo salar* tac3a (XP_013991334.1), *Oncorhynchus mykiss* tac3a (XP_021421533.1), *Engraulis mordax* tac3a (DAA35163.1), *Oryzias latipes* tac3a (NP_001265832.1), *Danio rerio* tac3a (NP_001243318.1), *Ictalurus punctatus* tac3a (XP_017343311.1), *Danio rerio* tac3b (NP_001243319.1), *Dicentrarchus labrax* tac3b (QYB17058.1), *Salmo salar* tac3b (XP_014022643.1), *Oncorhynchus mykiss* tac3b (CDQ91399.1), *Gallus gallus* tac3 (XP_015155892.2), *Nomascus leucogenys* tac3 (XP_003252831.1), *Macaca mulatta* tac3 (XP_001115535.2), *Homo sapiens* tac3 (AAQ10783.1), *Canis lupus familiaris* tac3 (NP_001279988.1), *Sus scrofa* tac3 (NP_001007197.1), *Rattus norvegicus* tac2 (NP_062035.1), *Mus musculus* tac2 (BAA03316.1), *Takifugu rubripes* tacr3a1 (NP_001267026.1), *Tetraodon nigroviridis* tacr3a1 (DAA35148.1), *Oryzias latipes* tacr3a1 (NP_001265839.1), *Gasterosteus aculeatus* tacr3a1 (XP_040043470.1), *Takifugu rubripes* tacr3a2 (NP_001267041.1), *Tetraodon nigroviridis* tacr3a2 (DAA35149.1), *Oryzias latipes* tacr3a2 (NP_001265803.1), *Gasterosteus aculeatus tacr3a2* (XP_040042202.1), *Danio rerio* tacr3a1 (NP_001243567.1), *Danio rerio* tacr3a2 (NP_001243564.1), *Danio rerio* tacr3b (XP_002666594.1), *Homo sapiens* tacr3 (NP_001050.1), *Mus musculus* tacr3 (AAH66845.1), *Rattus norvegicus* tacr3 (NP_058749.1), *Mus musculus* tacr1 (NP_033339.2), *Rattus norvegicus* tacr1 (NP_036799.1), *Homo sapiens* tacr1 (NP_001049.1), *Gallus gallus* tacr1 (NP_990199.1),*Xenopus tropicalis* tacr1 (NP_001106489.1), *Takifugu rubripes* tacr1b (XP_029693280.1), *Tetraodon nigroviridis* tacr1b (CAG05392.1), *Oryzias latipes* tacr1b (XP_011479687.1), *Danio rerio* tacr1b (NP_001268728.1), *Danio rerio* tacr1a (NP_001257407.1), *Oryzias latipes* tacr1a (NP_001265826.1), *Gasterosteus aculeatus* tacr1a (XP_040052094.1), *Takifugu rubripes* tacr1a (NP_001267036.1), *Tetraodon nigroviridis* tacr1a (CAG12579.1), *Homo sapiens* tacr2 (NP_001048.2), *Mus musculus* tacr2 (NP_033340.3), *Danio rerio* tacr2 (NP_001314788.1), *Gasterosteus aculeatus* tacr2 (XP_040059362.1), *Oryzias latipes* tacr2 (XP_011489426.1), *Takifugu rubripes* tacr2 (NP_001267009.1), and *Tetraodon nigroviridis* tacr2 (DAA35150.1).

Phylogenetic trees of the TAC3s and TACRs were constructed. As shown in **Figure 3A**, TAC3s from teleost and other vertebrates formed two large clades, and the teleost clade was subdivided into TAC3a and TAC3b clades. The two TAC3 of the Japanese eel are clustered in the TAC3b clade, which is clustered together with the TAC3b of Atlantic salmon (*Salmo salar*), rainbow trout (*Oncorhynchus mykiss*), and European sea bass (*Dicentrarchus labrax*) TAC3b. Phylogenetic tree of TACRs showed three TACR clades, which Japanese eel TACR3a was clustered into TACR3 clade, indicating the evolutionary conservation (**Figure 3B**).

### Tissue Expression of Japanese Eel *tac3*s

**Figure 4:**
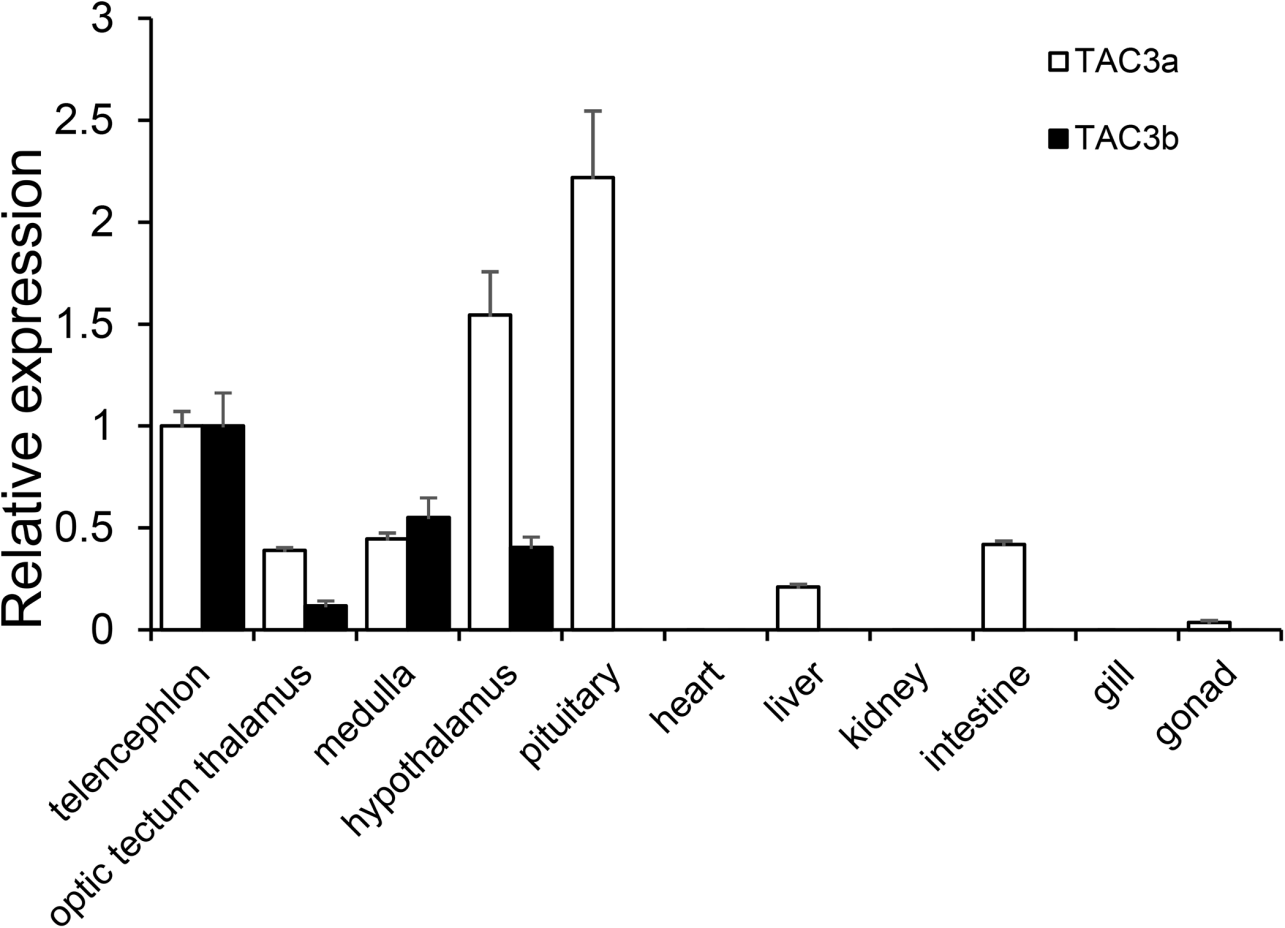
Expression of *tac3s* mRNAs in various tissues (telencephalon, optic tectum thalamus, medulla, hypothalamus, pituitary, heart, liver, kidney, intestine, gill, and gonad) of Japanese eel. The X-axis indicates different tissues. The results are normalized to 18s rRNA. The data are shown as the mean ± S.E.M (n = 3), and the mRNA levels of *tac3a* and *tac3b* were calculated as fold change relative to the mRNA levels of the heart. The different lowercased letters indicated significant differences (P < 0.05).

The expression patterns of the *tac3*s genes in different tissues and brain regions were shown in **Figure 4**. *Tac3a* mRNA was mainly expressed in the pituitary, followed by the hypothalamus and telencephalon, with low level expressed in the optic tectum, medulla, liver, intestine, and gonad. However, *tac3b* mRNA was only expressed in telencephalon, optic tectum, medulla, and hypothalamus, and the expression was relatively high in the telencephalon.

### Localization of the mRNA of *tac3s* in the Brain

**Figure 5:**
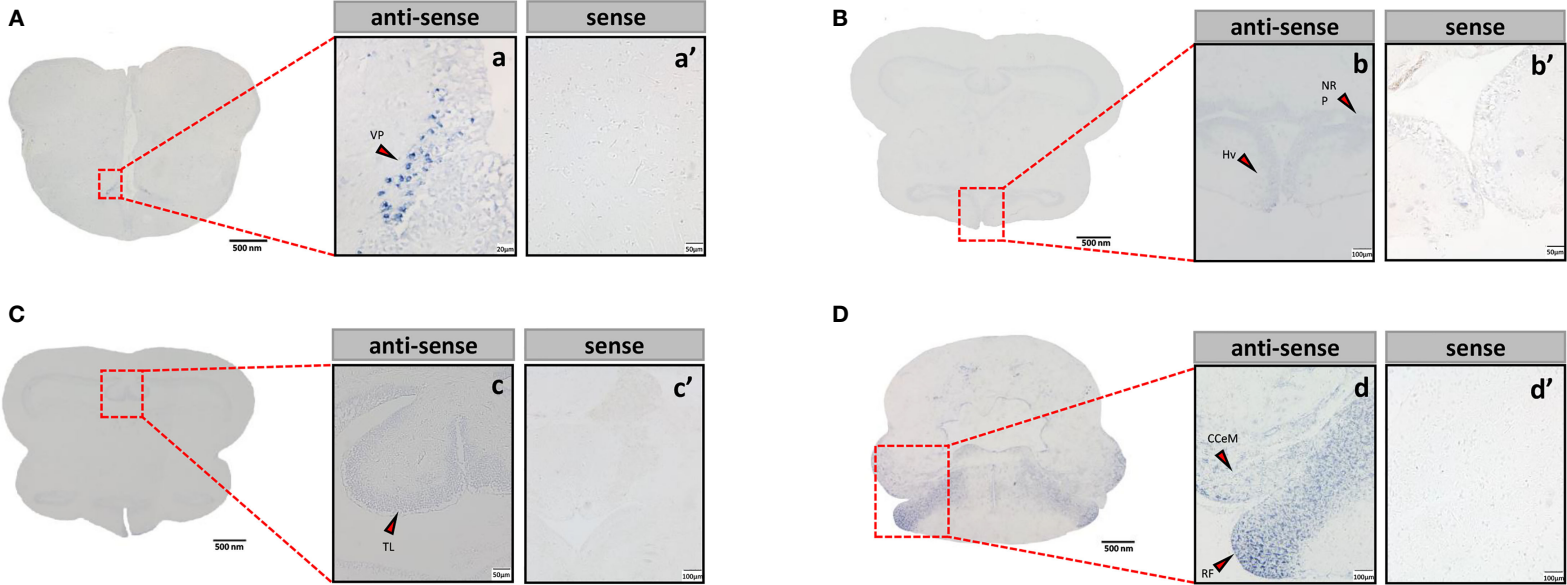
*Tac3a* mRNA in the brain of Japanese eel was detected by *in situ* hybridization (ISH). *Tac3a* localization in the telencephalon **(A)**, thalamus **(B)**, midbrain **(C)**, and cerebellum **(D)**. Scale bar = 500 μm. Positive signal of *tac3a* in the telencephalon **(a)**, thalamus **(b)**, midbrain **(c)**, and cerebellum **(d)**. Negative signals of *tac3a* with sense probe results in the telencephalon **(a’)**, thalamus **(b’)**, midbrain **(c’)**, and cerebellum **(d’)**. b, c’, d, d’: Scale bar = 100 μm. a’, b’, c: scale bar = 50 μm. a: scale bar = 20 μm. VP, the postcommissural nucleus of the ventral telencephalon; Hv, the ventral hypothalamus; NRP, the nucleus of the posterior recess; TL, the torus longitudinalis; RF, the reticular formation; CCeM, the molecular layer of corpus cerebelli.

**Figure 6:**
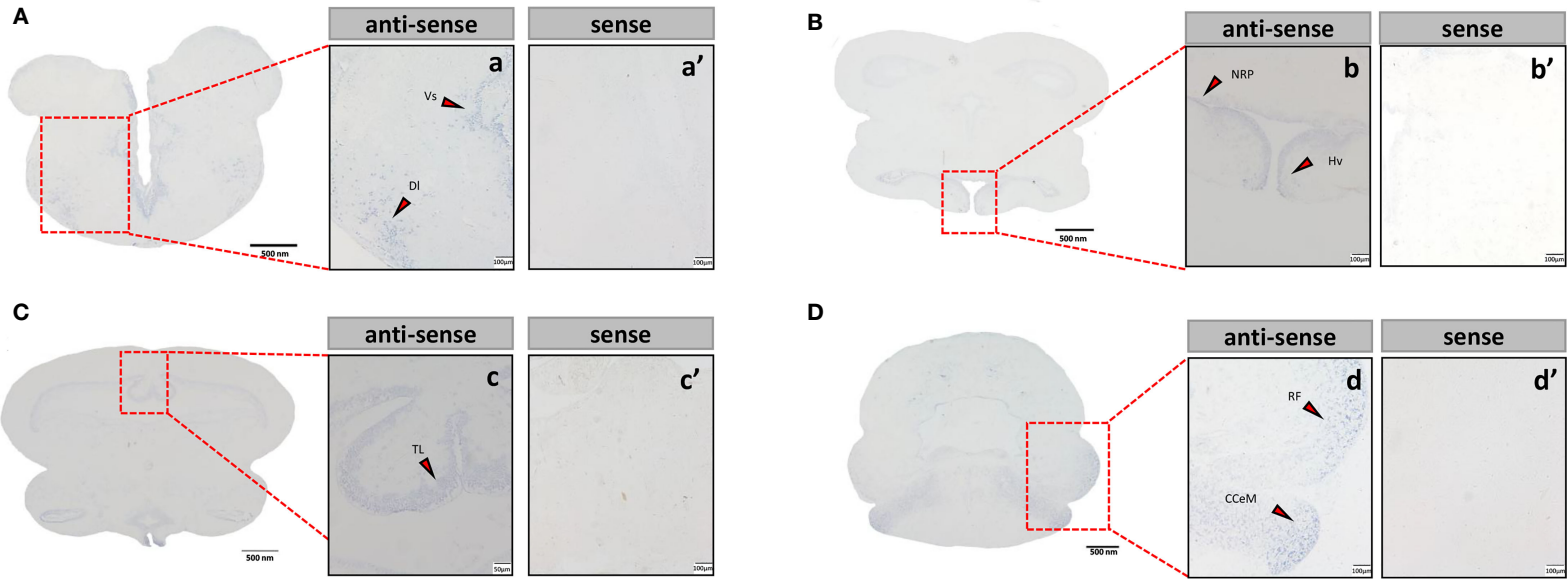
*Tac3b* mRNA in the brain of Japanese eel was detected by *in situ* hybridization (ISH). *Tac3b* localization in the telencephalon. *Tac3b* localization in the telencephalon **(A)**, thalamus **(B)**, midbrain **(C)**, and cerebellum **(D)**. Scale bar = 500 μm. Positive signal of *tac3b* in the telencephalon **(a)**, thalamus **(b)**, midbrain **(c)**, and cerebellum **(d)**. Negative signals of *tac3a* with sense probe results in the telencephalon **(a’)**, thalamus **(b’)**, midbrain **(c’)**, and cerebellum **(d’)**. a, a’, b, b’, c’, d, d’: Scale bar = 100 μm. c: scale bar = 50 μm. Dl, the lateral part of the dorsal telencephalon; Vs, the supracommissural nucleus of the ventral telencephalon; Hv, the ventral hypothalamus; NRP, the nucleus of the posterior recess; TL, the torus longitudinalis; RF, the reticular formation; CCeM, the molecular layer of corpus cerebelli.

Sections of brain tissue of Japanese eel were taken to detect the mRNA distribution of *tac3a* and *tac3b* by *in situ* hybridization. Localization of both *tac3a* and *tac3b* in the brain is shown in **Figures 5**, **6**. *Tac3a* positive signals were detected in the postcommissural nucleus of the ventral telencephalon (VP), the ventral hypothalamus (Hv), the nucleus of the posterior recess (NRP), the torus longitudinalis (TL), the reticular formation (RF), the molecular layer of corpus cerebelli (CCeM) (**Figures 5A–D**). The *tac3b* positive signals were detected in the lateral part of the dorsal telencephalon (Dl), the supracommissural nucleus of the ventral telencephalon (Vs), the Hv, the NRP, the TL, the RF, and the CCeM (**Figures 6A–D**).

### Binding Analysis of NKB and TACR3 in Japanese Eel

**Figure 7:**
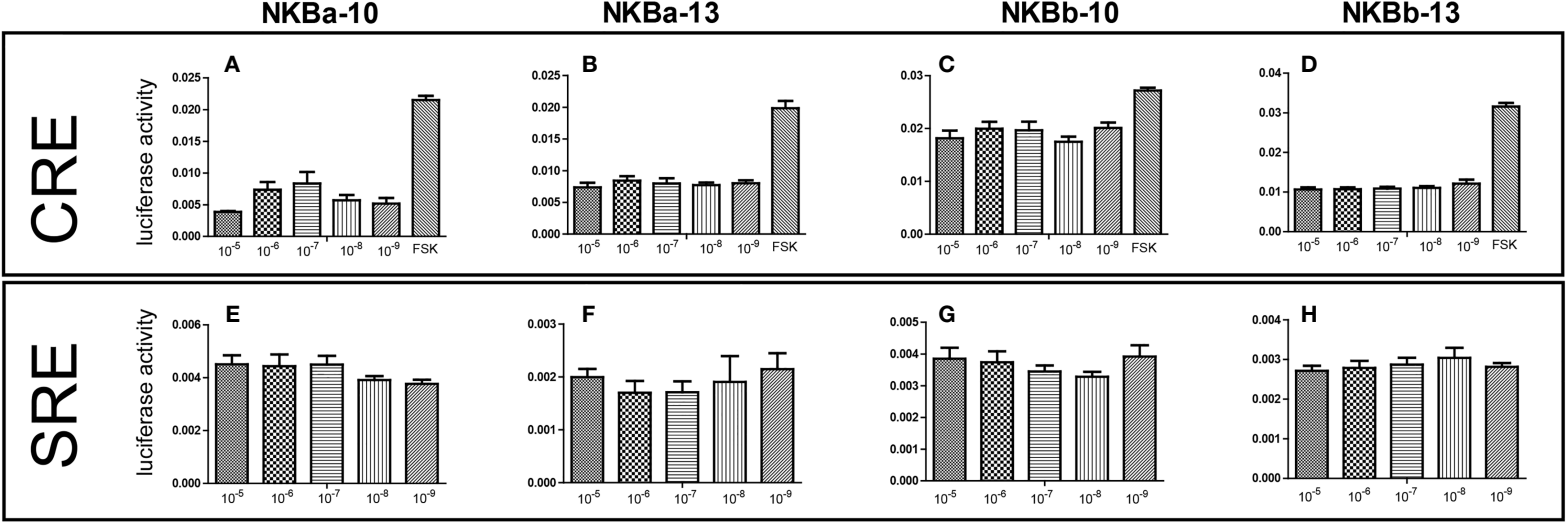
**(A–D)** NKB peptides induced CRE driven promoter activities co-transfected with Japanese eel *tacr3*. **(E–H)** NKB peptides induced SRE driven promoter activities co-transfected with Japanese eel *tacr3*.

**Figure 8:**
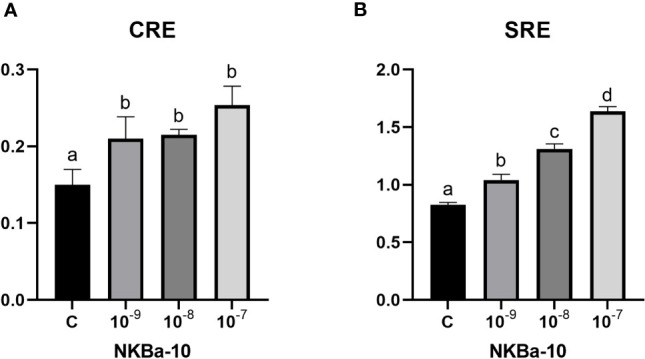
**(A)** NKBa-10 peptides induced CRE driven promoter activities co-transfected with zebrafish *tacr3a1*. **(B)** NKBa-10 peptides induced SRE driven promoter activities co-transfected with zebrafish *tacr3a1*. The different lowercased letters indicated significant differences (P < 0.05).

As shown in **Figure 7**, NKBa-10 of Japanese eel could weakly activate the CRE pathway *via* TACR3, whereas NKBa-13, NKBb-10, and NKBb-13 had no effect under the same conditions. Moreover, none of the four peptides binding with the receptor TACR3 could activate the SRE pathway, which indicated that they could not cause the increase of transcriptional activity of SRE pathway. In **Figure 8**, NKBa-10 of Japanese eel binding with TACR3A1 receptor of zebrafish could effectively activate CRE and SRE signaling pathways, especially significantly increasing the transcriptional activity of SRE signaling pathway.

### Regulation of mRNA Levels of *mgnrh*, *cgnrh*, *lhb*, and *fshb* genes in the Brain by NKB *in vivo*

**Figure 9:**
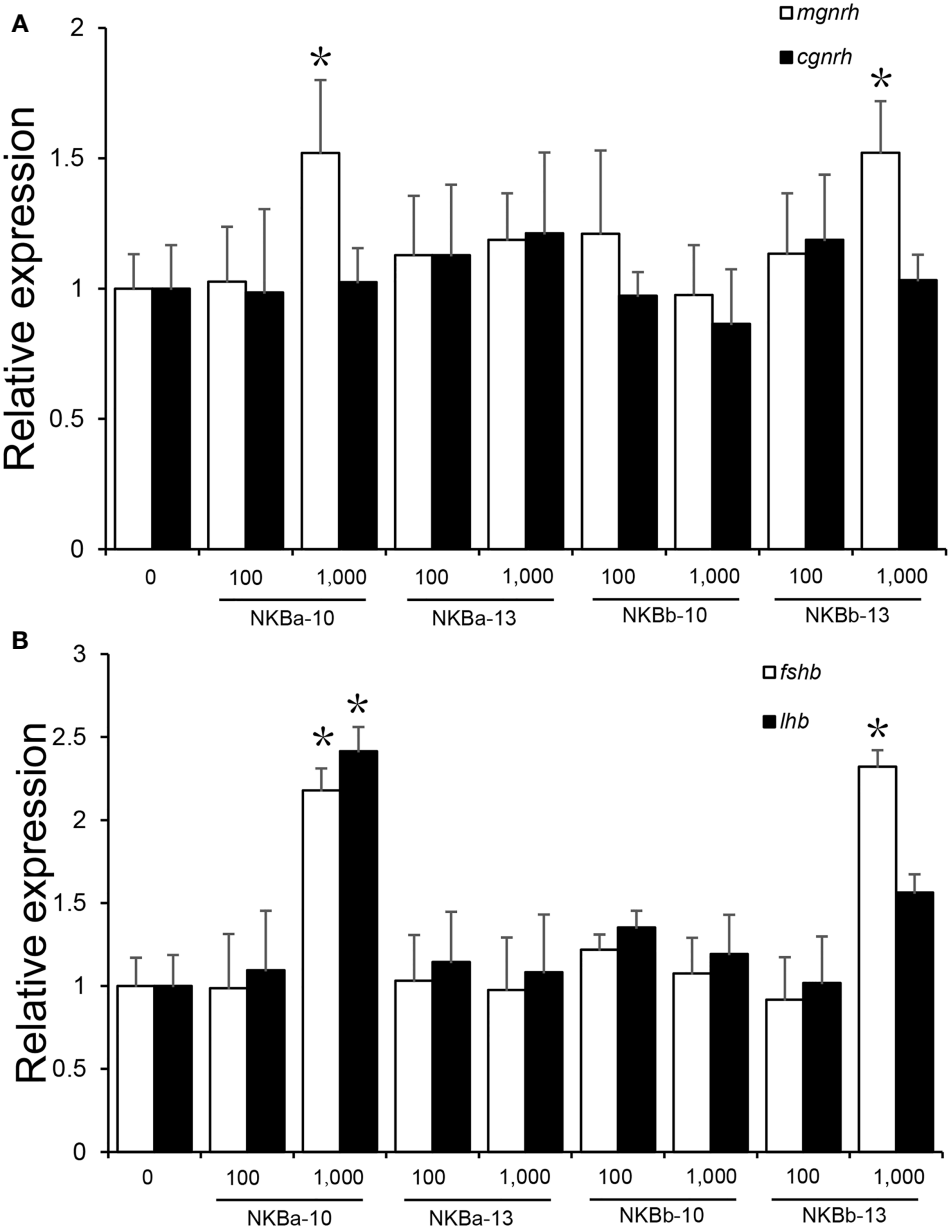
**(A)** Effects of NKBa-10, NKBa-13, NKBb-10, and NKBb-13 on *mgnrh* and *cgnrh* expression of Japanese eel after intraperitoneal injection for 6 h. The X-axis indicates the different NKB peptides and their corresponding concentrations. **(B)** Effects of NKBa-10, NKBa-13, NKBb-10, and NKBb-13 on *fshb* and *lhb* of Japanese eel after intraperitoneal injection for 6 h. The X-axis indicates the different NKB peptides and their corresponding concentrations. The asterisk indicated significant difference of one gene among different stimulate concentration (*P <* 0.05).

The results showed that high concentration (1,000 ng/g BW) of NKBa-10 and NKBb-13 could increase the expression level of *mgnrh* significantly (*P <* 0.05), whereas NKBa-13 and NKBb-10 had no effect on the expression of *mgnrh*. However, neither of the four peptides in low concentration (100 ng/g BW) had effect on the expression of *mgnrh*. In addition, both high and low concentration of NKBa-10, NKBa-13, NKBb-10, and NKBb-13 were unable to activate *cgnrh* expression compared with control group (**Figure 9A** **)**.

As shown in **Figure 9B**, high concentration (1,000 ng/g BW) of NKBa-10 and NKBb-13 could significantly upregulate the expression level of *fshb* (*P <* 0.05), whereas NKBa-13 and NKBb-10 had no effect on *fshb* in both peptide concentrations. In the regulation of *lhr*, only high concentration of NKBa-10 could increase *lhr* level significantly (*P <* 0.05). The other three peptides—NKBa-13, NKBb-10, and NKBb-13—could not regulate *lhr* level in both concentrations compared with control group.

## Discussion

Tachykinin, encoded by *tac1*, *tac3*, and *tac4*, is considered as one of the largest families of neuropeptides. It is named due to the ability to rapidly induce intestine contraction (32). More and more evidence showed that *tac1* gene produces substance P (SP) and neurokinin A (NKA), *tac3* encodes NKB and NKF, and *tac4* encodes hemokinin-1 (HK-1) and endokinins (33). Among them, NKB showed its essential role in the onset of puberty and gonadotropin secretion (22, 34). Different from mammals, with only one *tac* gene to code NKBs, teleosts undergo a teleost-specific genome duplication event (35), which, in the present, causes two *tac3* genes. In recent years, an increasing number of studies have reported that the NKB/NKR system plays a critical role in teleost, especially in reproduction (23, 36, 37). Previous studies also have reported two *tac3* genes (*tac3a* and *tac3b*) in zebrafish, goldfish, salmon, and spotted sea bass (22, 29, 36, 37). In the present study, we have identified two *tac3* and one *tacr3* genes in Japanese eel. There were two tachykinin peptides in each of Japanese eel TAC3 precursor including NKB-13 (also known as NKB-related peptide) and NKB-10, which suggested conserved functions in teleost.

Studies in mammals and other vertebrates showed that tachykinin shared conservative carboxyl-terminal sequence, -FXGLM-NH_2_ (38, 39). The four NKBs of Japanese eel generated from two *tac3* genes presented the common C-terminal motif (-FVGLM-NH_2_), which is also found in zebrafish (22), goldfish (23), and spotted sea bass (29). From a chemical point of view, two distinct moieties were presented in tachykinins: a N-terminal sequence with variability in all tachykinins and a C-terminal sequence, which is responsible for receptor activation (38). The terminal Met is regard as an essential factor for tachykinin function because the tachykinin-like peptides from invertebrates with a C-terminal Arg are unable to activate mammalian tachykinin receptors (40, 41). In the present study, three out of four NKBs in Japanese eel—NKBa-13, NKBa-10, and NKBb-13—had identical C-terminal Met, which is supported by the results in zebrafish (22), goldfish (23), spotted sea bass (29), and European eel (*Anguilla anguilla*) (42).

In the present study, we found mutation in *tacr3*. To confirm whether the mutation is ubiquitous or from an individual difference, we have collected more than 80 Japanese eels both cultured and wild caught from different locations, including Guangzhou (113°E, 23.5°N), Zhuhai (Guangdong) (113.5°E, 22°N), and Qingdao (Shandong) (120.4°E, 36°N), China. The DNA was purified for PCR with specific primers to amplified the mutation site. The PCR products were then subcloned into *E. coli*, and three clones were sent for Sanger’s sequencing to confirm the accuracy. As a result, all the sequences confirmed the mutation. In addition, we have noticed the European eel (*Anguilla Anguilla*) TAC3R sequence (XM_035417067.1), when the mutation in Japanese eel was confirmed. However, the sequence is predicted on the basis of the whole genome sequence. Hence, we have further checked the whole genome sequences in both European eel and American eel (*Anguilla rostrata*). Interestingly, both genome sequences showed the same results with the Japanese eel, which ended at the mutation site. Unfortunately, we are not able to get the sample or DNA of these eels, so we can not 100% sure about the mutation in other eel species. However, we can be sure about the mutation in the Japanese eel.

Tissue distribution results showed that both *tac3a* and *tac3b* were highly expressed in the brain of Japanese eel, consistent with the results in other fishes including zebrafish, goldfish, orange-spotted grouper (*Epinephelus coioides*), and spotted sea bass (22, 23, 26, 29, 37, 43). Furthermore, *tac3a* was found mainly expressed in the hypothalamus and pituitary. It is proverbial that the hypothalamus plays an important role in reproductive regulation, which suggests that *tac3s* genes may be involved in reproductive regulation of Japanese eel as well. In zebrafish and spotted sea bass, *tac3a* was mainly expressed in the hypothalamus but not detected in the pituitary (22, 29, 37). Whereas, in goldfish, tac3a was strongly expressed in the pituitary (23), consistent with the result in Japanese eel. These results indicated that the expression pattern may be variable in different species. The expression of *tac3b* in Japanese eel was detected only in the brain but not in pituitary, which is coincident with the result in goldfish (13). In gonad, *tac3b* was not detected in Japanese eel, which is different from zebrafish or spotted sea bass (29, 37), which may be caused by the different developing stages of gonad.

*In situ* hybridization results showed that *tac3*s positive signals were observed in multiple brain regions including telencephalon, mesencephalon, and hypothalamus, which were similar to the results in spotted sea bass (29) and orange-spotted grouper (36). Although it has been reported that *tac3*s expression is different in brain regions among different species, which is speculated to be caused by interspecies difference or physiological state difference (29). However, *tac3s* expression was still highest in the brains of teleosts. In addition, it has been established that NKBs is a key regulator of GnRH, regulating the onset of puberty and reproductive physiology in mammals (44), and is involved in the regulation of the reproductive axis in some teleosts, which suggests that NKBs may be involved in the reproductive process and have regulatory effect.

To test whether the mutation in *tacr3* cause intracellular structural incompletion, we synthesized four NKBs and used luciferase assay to detect the activation of CRE and SRE signaling pathways. Our results showed that none of the NKBs could activate neither CRE nor SRE signaling pathway *via* Japanese eel TACR3. In addition, we conducted experiments using NKBa-10 through zebrafish receptor and found that it could activate both two signaling pathways effectively. As a result, the Japanese eel receptor is dysfunctional in further signal transduction (45, 46). By blasting the genome sequence, we figured out another potential tac3r; however, limited to the yellow eel samples, the cloning of the gene failed. These results indicate a possibility that the transcription of the other tac3r was started when the yellow eel turned to the silver eel. A large number of studies proved that mutations in the *tac3* or *tacr3* genes lead function decline at the hypothalamic level (47). In different species, NKB has different regulatory effects on the release of LH, and the similar condition occurs in different developmental stages of the same species. For example, NKB has no effect on the release of LH in luteinized ewes, but it can promote the release of LH in follicular phase (48). We investigated the reproductive regulation of NKB in Japanese eel. Quantitative Real-time (qPCR) results showed that NKBa-10 and NKBb-13 in high concentration can upregulate the mRNA levels of *mgnrh* and *fshb* of Japanese eel, whereas only high concentration of NKBa-10 can increase the mRNA levels of *lhb*; taking the luciferase assay and the *in vivo* injection results together, the NKBs may bind to other receptors to increase the *gnrh* or *gth* expression level.

In conclusion, our present study provides the novel insights into the reproductive function of NKB in Japanese eel. We identified and characterized the NKB/NK3R system in Japanese eel. In addition, we demonstrated dysfunction of the TAC3R and the stimulation of *gnrh* and *gth* by high dose of NKBs. These results present the first demonstrates of NKB/NK3R system in reproduction of Japanese eel.

## Data Availability Statement

The datasets presented in this study can be found in online repositories. The names of the repository/repositories and accession number(s) can be found below: NCBI; OL804257.1, OL804258.1.

## Ethics Statement

The animal study was reviewed and approved by Animal Research and Ethics Committees of Sun Yat-sen University and Ocean University of China.

## Author Contributions

XQ, HW, and YL designed the study. WZ performed the RNA extraction and cDNA preparation. WZ performed the sequence analysis and the tissue expression analysis. CZ performed the *in situ* hybridization (ISH) experiment. WZ performed cell culture and co-transfection. WZ performed the *in vivo* injection. CZ and LL wrote the manuscript. XQ provided manuscript editing and feedback. All authors read and approved the final manuscript.

## Funding

This study was supported by grants from the National Natural Science Foundation of China (41976089).

## Conflict of Interest

The authors declare that the research was conducted in the absence of any commercial or financial relationships that could be construed as a potential conflict of interest.

## Publisher’s Note

All claims expressed in this article are solely those of the authors and do not necessarily represent those of their affiliated organizations, or those of the publisher, the editors and the reviewers. Any product that may be evaluated in this article, or claim that may be made by its manufacturer, is not guaranteed or endorsed by the publisher.
